# A Comprehensive Overview of Skin Complications in Diabetes and Their Prevention

**DOI:** 10.7759/cureus.38961

**Published:** 2023-05-13

**Authors:** Pascaline David, Seema Singh, Ruchira Ankar

**Affiliations:** 1 Medical Surgical Nursing, Smt. Radhikabai Meghe Memorial College of Nursing, Datta Meghe Institute of Higher Education and Research, Wardha, IND

**Keywords:** topical agents, foot care, skin care, skin complication, skin problems, diabetes prevalence, diabetes

## Abstract

Diabetes is a widespread noncommunicable disease affecting millions of people globally, and it is accompanied by various complications ranging from minor to major. Among the complications, skin problems are highly prevalent in diabetic patients, including dry skin, itching, redness, scarring, and edema. Proper skin care is vital in preventing skin-related complications in diabetes. We conducted a comprehensive search of PubMed, Scopus, Google Scholar, and Web of Science databases for literature published from 2012 to 2022 using the following keywords: diabetes, diabetes prevalence, diabetes complications, skin problems in diabetes, and skin care protocols. Topical agents have been found effective in managing pruritus, xerosis, and other complications associated with diabetes. Skin care, especially foot care, is crucial in diabetes. Emollients and urea-based creams are commonly used for foot care. The review highlights the importance of following a skin care protocol to prevent skin-related complications in diabetes. Topical agents, emollients, and foot care are crucial in managing diabetic skin problems. Clinicians should ensure that patients with diabetes are educated about skin care's importance and provided with appropriate guidance to maintain healthy skin.

## Introduction and background

Diabetes is a chronic metabolic disorder characterized by high blood glucose levels resulting from the body's inability to produce or effectively use insulin. It is a growing public health concern globally, affecting an estimated 463 million adults worldwide. In India, diabetes is also a major health problem, with an estimated 77 million adults diagnosed with the disease, and Maharashtra is one of the states with the highest prevalence [1].

One of the many complications of diabetes is skin complications. Skin complications in diabetes can affect up to 80% of patients and have significant consequences for a patient's quality of life. In addition, these complications can result in increased morbidity and mortality rates. Common skin complications of diabetes include diabetic dermopathy, skin infections, and xerosis [2].

The incidence of diabetes mellitus in India is reaching epidemic proportions, with an alarming increase in morbidity and mortality rates due to its potential complications. This disease poses a significant healthcare burden on families and society, with implications that extend to the broader economy. Notably, diabetes is now observed in younger individuals and is associated with a spectrum of complications. The steady migration of people from rural to urban areas, the economic boom, and consequent changes in lifestyle all contribute to the rising prevalence of diabetes in India. However, despite the evident increase in diabetes, there remains a paucity of studies investigating the precise status of the disease, partly because of the geographical, socio-economic, and ethnic diversity of the country. Given the high visibility of this disease across all strata of Indian society, there is an urgent demand for research and intervention at both regional and national levels to mitigate the potentially catastrophic increase in diabetes predicted for the coming years [3].

Given the high prevalence of diabetes and its associated skin complications, it is crucial to address this issue and explore strategies for its prevention and management. Early detection and treatment of skin complications can improve outcomes and reduce the risk of serious complications. Thus, this review article aims to provide a comprehensive overview of skin complications in diabetes, including their epidemiology, pathophysiology, and risk factors. In addition, we review current guidelines and protocols for skin care in diabetes, including the role of emollients and other topical treatments.

## Review

Prevalence and risk factors of diabetes mellitus

Diabetes is a chronic metabolic disorder characterized by high blood glucose levels, which can lead to various complications.

Incidence and Prevalence of Diabetes

According to the International Diabetes Federation, the global prevalence of diabetes among adults aged 20-79 years was estimated to be 9.3% in 2019, affecting approximately 463 million people. In India, the prevalence of diabetes has been steadily increasing over the past few decades, with an estimated 77 million adults diagnosed with the disease. Maharashtra is one of the states with the highest prevalence of diabetes in India, with an estimated 16 million people affected by the disease [4].

The impact of lifestyle choices and climate change is now evident in the evolving disease profile of individuals, with a substantial shift from communicable to non-communicable diseases. According to the National Health Profile 2019, the prevalence of lifestyle-based diseases such as diabetes, hypertension, stroke, and cancer is rising. Notably, the state of Maharashtra has a higher incidence of hypertension and diabetes patients than the national average. The Ministry of Health has reported that Maharashtra is one of the top three states in India for screening citizens for non-communicable diseases. Between January and December 2018, 5,883,915 individuals were screened for non-communicable diseases in the state, ranking second only to Tamil Nadu. Of these individuals, 1.55 lakh were diagnosed with diabetes, 2.50 lakh with hypertension, nearly 1 lakh with both hypertension and diabetes, and 14,000 with various types of cancer [5].

Risk Factors for Diabetes

There are several risk factors associated with the development of diabetes. These include genetics, age, physical inactivity, poor diet, and obesity. Other risk factors may include gestational diabetes, a family history of diabetes, and certain medical conditions such as high blood pressure and high cholesterol [6].

Common Types of Diabetes

There are several types of diabetes, but the most common are type 1 diabetes, type 2 diabetes, and gestational diabetes. Type 1 diabetes is an autoimmune disorder that typically develops in childhood or adolescence and is characterized by the destruction of insulin-producing beta cells in the pancreas. Type 2 diabetes is the most common type of diabetes and is typically associated with lifestyle factors such as poor diet and lack of exercise. Gestational diabetes is a type of diabetes that develops during pregnancy and typically resolves after delivery but may increase the risk of developing type 2 diabetes later in life [7].

Complications of the diabetes mellitus

Diabetes mellitus is a chronic metabolic disorder characterized by elevated blood glucose levels resulting from the inability of the body to produce or effectively use insulin. While the diagnosis of diabetes is concerning in and of itself, it is further complicated by a range of potential complications that can significantly impact the health outcomes of those with the disease [8].

Among the complications associated with diabetes, microvascular and macrovascular complications are the most commonly observed. Microvascular complications involve damage to the small blood vessels throughout the body, leading to diabetic retinopathy, diabetic nephropathy, and diabetic neuropathy. Diabetic retinopathy is the leading cause of blindness in working-age adults, while diabetic nephropathy can lead to end-stage renal disease. Diabetic neuropathy, on the other hand, can result in severe pain, numbness, and tingling sensations in the extremities [9].

Macrovascular complications of diabetes, on the other hand, affect the larger blood vessels and can include conditions such as coronary artery disease, stroke, and peripheral vascular disease. Coronary artery disease, a major cause of morbidity and mortality in patients with diabetes, occurs when the blood vessels supplying the heart become narrowed or blocked, leading to angina, myocardial infarction, or heart failure. On the other hand, peripheral vascular disease can cause pain, numbness, and ulcers in the legs and feet and may result in the need for amputation [10].

Risk Factors for Complications

The development of complications in diabetes is known to be associated with several risk factors. Among these, poor blood glucose control, high blood pressure, and high cholesterol are major contributors to the onset of complications. Smoking, obesity, and physical inactivity have also been linked to an increased risk of developing complications in diabetes [11,12].

Furthermore, certain genetic factors and the duration of diabetes are also known to play a crucial role in determining the risk of complications in diabetes. Moreover, the presence of other medical conditions, such as kidney disease and cardiovascular disease, may exacerbate the development of complications in diabetes [13].

Overall, understanding the various risk factors associated with the development of complications in diabetes is critical for healthcare professionals to devise effective preventive and management strategies for this chronic condition.

Implications of Uncontrolled Diabetes on Health Outcomes

Uncontrolled diabetes poses a grave threat to health outcomes, as it can lead to debilitating complications with far-reaching consequences. These complications can manifest in diverse forms, such as blindness, kidney failure, amputations, heart disease, and stroke. The deleterious effects of uncontrolled diabetes can also extend to a heightened susceptibility to infections and mental health disorders. Consequently, addressing and managing diabetes and its associated complications is critical for averting these adverse health outcomes. Failure to control diabetes can exacerbate the risk of morbidity and mortality, causing undue burden on patients, their families, and the healthcare system as a whole. As such, there is a pressing need for effective preventive measures and therapeutic interventions aimed at mitigating the complications of diabetes and improving health outcomes for individuals affected by this disease [14,15].

Skin complications in diabetes

Diabetes is a chronic condition that affects the body's ability to regulate blood sugar levels. It can lead to various complications, including several skin-related issues. Here are some common skin complications that can occur in diabetes:

Diabetic Dermopathy

Diabetic dermopathy is a skin condition commonly observed in individuals with diabetes. It is also known as shin spots because it frequently appears on the lower legs, specifically the shins. The condition is characterized by light brown or red scaly patches on the skin, which may be slightly raised or depressed [11].

The exact cause of diabetic dermopathy is unknown, but it is believed to be related to microvascular complications due to long-term high blood sugar levels. These complications can damage the small blood vessels in the skin, leading to the formation of lesions. The patches of diabetic dermopathy are typically asymptomatic, which means they do not cause pain or itching. However, they can be slow to heal if injured, as the damaged blood vessels may not be able to deliver nutrients and oxygen to the affected area effectively [12].

Diabetic dermopathy is not a serious condition and does not require any specific treatment. The patches may fade, but they can persist for years in some individuals. Although the condition is harmless, it may cause distress for some people, particularly if the patches are visible on the legs. To prevent diabetic dermopathy, individuals with diabetes should strive to maintain good blood sugar control. They should also protect their skin from injury by wearing protective footwear and avoiding exposure to extreme temperatures [13].

Diabetic Blisters (Bullosis Diabeticorum)

Diabetic blisters, also known as bullosis diabeticorum, are relatively uncommon skin complications that can occur in individuals with diabetes. These blisters are characterized by fluid-filled sacs that develop on various body parts, including the legs, feet, hands, and arms [11]. One distinguishing feature of diabetic blisters is their size. They tend to be larger than typical blisters and can range from a few millimeters to several centimeters in diameter. Despite their significant size, these blisters are painless, differentiating them from other types of blisters that may cause discomfort or tenderness. The exact cause of diabetic blisters is not yet fully understood, but several factors may contribute to their development. One possible explanation is that long-standing uncontrolled blood sugar levels can lead to nerve damage (diabetic neuropathy). This neuropathy can affect the sensation in the skin, making it more susceptible to trauma or friction. In some cases, even minor injuries or repetitive friction from activities such as walking or wearing ill-fitting shoes can trigger the formation of blisters. Unlike typical blisters that often result from burns or intense friction, diabetic blisters tend to appear spontaneously and without an obvious cause. They may suddenly emerge on seemingly unaffected skin. It's important to note that these blisters are not contagious or caused by an infection [12-14].

Fortunately, diabetic blisters usually resolve independently within a few weeks, even without specific treatment. The fluid inside the blisters will gradually be reabsorbed by the body, and the blisters will dry up and heal. However, it is important not to puncture or forcefully rupture the blisters, as this can increase the risk of infection. While the blisters themselves are harmless, their presence may indicate underlying complications related to diabetes. For instance, developing blisters can indicate poor blood sugar control, peripheral neuropathy, or other vascular issues. Therefore, it is crucial for individuals with diabetes who experience diabetic blisters to consult with a healthcare professional. The healthcare provider can assess the overall condition, provide appropriate guidance for managing blood sugar levels, and monitor for any signs of complications [13].

Digital Sclerosis

Digital sclerosis is a condition that affects the skin and joints of the fingers, toes, and hands in individuals with diabetes. It is characterized by the thickening and tightening of the skin in these areas, leading to a waxy appearance and texture. The exact cause of digital sclerosis is not fully understood, but it is believed to be associated with long-term exposure to high blood sugar levels and poor circulation [12].

One of the primary symptoms of digital sclerosis is the thickening of the skin. The skin may become hardened and lose its normal elasticity, resulting in a tight and shiny appearance. The affected areas can feel stiff and inflexible. As the condition progresses, moving the fingers, toes, and hands may become increasingly difficult. This stiffness and limited mobility can significantly impact daily activities, such as writing, typing, gripping objects, or performing fine motor tasks. The joint stiffness associated with digital sclerosis can be particularly problematic. It may affect multiple joints, reducing the range of motion and flexibility. This can make it challenging to perform simple tasks that require finger and hand movements, such as buttoning a shirt or tying shoelaces. The stiffness and limited mobility in the fingers and toes can also affect balance and coordination [15-16].

Digital sclerosis can significantly impact a person's quality of life, affecting their ability to perform essential tasks and engage in activities they enjoy. The thickened and waxy skin is also more prone to cracking and developing open sores, increasing the risk of infections [14].

Diabetic Foot Ulcers

Diabetic foot ulcers are a serious complication that can arise due to the combination of high blood sugar levels, nerve damage (neuropathy), and poor blood circulation (peripheral arterial disease) commonly seen in diabetes [15].

Nerve Damage (Neuropathy)

Prolonged exposure to high blood sugar levels can damage the nerves, particularly the peripheral nerves that extend to the feet. This condition is known as peripheral neuropathy. Neuropathy can cause a loss of sensation in the feet, making it difficult for individuals to detect injuries, pressure points, or discomfort. As a result, minor injuries such as blisters, cuts, or ulcers may go unnoticed or be ignored due to the lack of pain [16].

Unnoticed Injuries or Sores

With reduced sensation in the feet, individuals with diabetic neuropathy may continue to walk or pressure areas injured or affected by excessive friction, such as ill-fitting shoes. These repetitive injuries can lead to the formation of blisters, calluses, or skin breaks. Since they are not immediately noticed or treated, they can worsen over time [17].

Poor Blood Circulation (Peripheral Arterial Disease)

Diabetes can also affect blood vessels, reducing blood flow and circulation in the lower extremities. This condition is known as peripheral arterial disease. Inadequate blood supply hinders the delivery of oxygen and nutrients necessary for wound healing. Consequently, even minor injuries take longer to heal and are at a higher risk of developing complications [18].

Development of Foot Ulcers

When injuries or pressure points persist without proper care and attention, they can progress to become foot ulcers. Foot ulcers are open sores or wounds that commonly occur on the bottom of the foot or pressure points such as the sides or tips of the toes. These ulcers can be deep and difficult to heal due to compromised blood flow and impaired healing mechanisms in diabetes [14].

Risk of Infection and Amputation

Diabetic foot ulcers have a high risk of becoming infected. The presence of an open wound provides an entry point for bacteria, which can multiply and cause a serious infection. Infections can spread rapidly, leading to cellulitis (skin infection), abscess formation, or osteomyelitis (bone infection). If the infection becomes severe and progresses, it can lead to tissue death (gangrene). It may necessitate amputation of the affected toe, foot, or even part of the leg to prevent the spread of infection [15].

To prevent diabetic foot ulcers and their complications, individuals with diabetes should pay close attention to foot care. This includes daily foot inspections, wearing well-fitting shoes, maintaining good blood sugar control, practicing proper foot hygiene, avoiding prolonged periods of sitting or standing, and seeking medical attention promptly for any signs of foot injury, infection, or non-healing wounds. Regular foot exams by a healthcare professional are also crucial for the early detection of any problems and appropriate management [16-17].

Infections

People with diabetes are more prone to skin infections due to several factors. High blood sugar levels weaken the immune system, making it difficult for the body to fight off infections. Additionally, diabetes can cause nerve damage, reducing the ability to feel pain, heat, or cold. This means that individuals with diabetes may not notice injuries or infections as easily as people without the condition [14].

Styes are bacterial infections that affect the eyelid, usually caused by *Staphylococcus aureus*. The bacteria can infect the eyelid's oil glands, leading to a red, swollen, and painful lump. If left untreated, a stye can grow and cause complications such as spreading the infection to other eye parts. People with diabetes are at higher risk of developing styes due to their weakened immune system [15].

Boils and carbuncles are deep skin infections caused by bacteria such as *Staphylococcus aureus*. They usually appear as painful, red, swollen bumps filled with pus. Boils and carbuncles can occur anywhere on the body, but they are most common on the face, neck, armpits, buttocks, and thighs. People with diabetes are more susceptible to these infections due to weakened immune systems and impaired blood flow to the skin [16].

Folliculitis is a bacterial or fungal infection of the hair follicles, which are small sacs surrounding hair roots. It usually appears as small red or white bumps that may be itchy or painful. Folliculitis can occur anywhere on the body but is most common on the face, scalp, arms, and legs. People with diabetes are more prone to folliculitis due to weakened immune systems and poor circulation [14].

UTIs are bacterial infections that affect the bladder, kidneys, ureters, or urethra. They are more common in people with diabetes due to high blood sugar levels, which can promote bacterial growth in the urinary tract. Symptoms of UTIs include pain or burning during urination, frequent urination, fever, and abdominal pain [16].

Fungal infections such as yeast infections and jock itch are also more common in individuals with diabetes. Yeast infections are caused by an overgrowth of the fungus Candida, which can affect various body parts, including the skin, mouth, throat, and genitals. Jock itch is a fungal infection that affects the groin area, causing redness, itching, and a rash. People with diabetes are more prone to these infections due to their weakened immune systems and high blood sugar levels, which can promote fungal growth [17].

Acanthosis Nigricans

Acanthosis nigricans is a skin condition that manifests as dark, thickened, and velvety patches of skin. These patches typically appear in body folds and creases, such as the neck, armpits, groin, and elbows. However, they can also affect other areas of the body [14].

The affected skin in acanthosis nigricans is usually tan or brown and has a velvety or velour-like texture. The patches may be flat or slightly raised and can vary in size and shape. The skin may sometimes become itchy, irritated, or develop an odor [15]. The most common underlying causes of acanthosis nigricans are insulin resistance and obesity. Insulin resistance occurs when the body's cells become less responsive to the effects of insulin, a hormone that regulates blood sugar levels. As a result, the body produces more insulin to compensate for the resistance. Insulin and insulin-like growth factor-1 (IGF-1) can stimulate the growth of skin cells and the production of melanin, leading to the development of acanthosis nigricans [13].

Obesity is closely linked to insulin resistance and is a significant risk factor for acanthosis nigricans. Excess body weight, particularly around the abdomen, can contribute to insulin resistance and the development of dark patches on the skin. Apart from insulin resistance and obesity, acanthosis nigricans can also be associated with other conditions such as polycystic ovary syndrome, certain medications (such as corticosteroids or oral contraceptives), hormonal disorders, and, in rare cases, certain types of cancer [16].

It is important to note that acanthosis nigricans is not harmful or contagious. However, it often serves as a visible indicator of an underlying health issue, particularly insulin resistance, and obesity, which are risk factors for type 2 diabetes. Therefore, if you notice any unusual changes in your skin, especially if they resemble acanthosis nigricans, it is recommended to consult with a healthcare professional for proper evaluation and to address any underlying health concerns. Treating acanthosis nigricans involves managing the underlying cause. This typically involves lifestyle modifications such as achieving a healthy weight through diet and exercise, improving insulin sensitivity through blood sugar control, and addressing any underlying medical conditions. In some cases, medications or topical treatments may be prescribed to help improve the appearance of the affected skin [17].

Regular monitoring and management of acanthosis nigricans and its underlying causes are important for maintaining overall health and reducing the risk of developing complications associated with insulin resistance and obesity, such as type 2 diabetes, cardiovascular disease, and metabolic syndrome [16]. Individuals with diabetes need to care for their skin to prevent complications properly. This includes maintaining good blood sugar control, practicing good hygiene, keeping the skin clean and moisturized, regularly checking the feet for any signs of injury or infection, and seeking medical attention for any concerning skin changes or problems. If you have diabetes and are experiencing any skin-related issues, it is recommended to consult with a healthcare professional for an accurate diagnosis and appropriate treatment [14].

Pathophysiology and risk factors for skin complications

The pathophysiology of skin complications in diabetes is complex and multifactorial. Changes in microvascular and macrovascular circulation, decreased immunity, and altered collagen synthesis can all contribute to developing skin complications. Risk factors for skin complications in diabetes include poor glycemic control, duration of diabetes, neuropathy, peripheral vascular disease, obesity, and smoking. Additionally, certain medications, such as corticosteroids, can increase the risk of skin complications in diabetes. Understanding the epidemiology, pathophysiology, and risk factors for skin complications in diabetes is crucial for developing effective prevention and management strategies [12,19,20].

Skin care in diabetes

Prevention and management of skin complications are essential for individuals with diabetes. Effective skin care in diabetes involves preventing, detecting, and managing skin complications. The primary goal of skin care in diabetes is maintaining the skin's integrity and preventing complications. This can be achieved through regular skin examinations, keeping the skin clean and dry, avoiding harsh soaps and detergents, and using appropriate moisturizers and emollients [21-23].

Current Guidelines and Protocols for Skin Care in Diabetes

Several prominent organizations, such as the American Diabetes Association and the International Diabetes Federation, have established comprehensive guidelines and protocols to ensure optimal management of skin complications in individuals with diabetes. These guidelines serve as essential resources for healthcare providers in delivering effective care and minimizing the risks associated with diabetes-related skin problems. The guidelines emphasize the importance of regular skin assessments as part of routine diabetes care. Healthcare providers are encouraged to conduct thorough examinations to identify any signs of skin complications promptly. Early detection can prevent the progression of issues and enable timely intervention [24].

Patient education plays a crucial role in these guidelines. It is recommended that healthcare providers educate individuals with diabetes about proper skin care practices. This includes emphasizing the importance of daily cleansing, moisturizing, and skin inspection. Patients should be educated on maintaining good hygiene, especially in areas prone to excessive moisture, such as the feet. Moisturization is a key component of skin care in diabetes. The guidelines highlight using suitable moisturizers and emollients to maintain skin integrity and prevent dryness. Regularly applying these products helps minimize adverse cutaneous events, such as cracking, itching, and skin infections. Emollients like urea or lactic acid are often recommended to enhance hydration and prevent excessive dryness [25].

Prompt intervention and treatment of skin complications are strongly advocated in the guidelines. This includes addressing common issues like bacterial and fungal infections, ulcerations, and other lesions. Healthcare providers are advised to prescribe appropriate antimicrobial therapies, dressings, or topical treatments based on the specific condition presented by the patient. In cases where skin complications are severe or unresponsive to standard therapies, referral to a dermatologist or wound care specialist is recommended. These specialists possess expertise in managing complex skin issues associated with diabetes. Collaboration with specialists ensures that patients receive specialized care and access to advanced treatment options when necessary. By adhering to these established guidelines and protocols, healthcare providers can play a critical role in reducing the burden of skin complications associated with diabetes. Implementation of regular skin assessments, patient education, appropriate moisturization, timely intervention, and referral when needed can significantly improve the overall quality of life for individuals with diabetes [24-25].

Role of Emollients and Topical Treatments in Skin Care

The role of emollients and topical treatments in skin care for individuals with diabetes cannot be overstated. Emollients, which function as moisturizers, are critical in hydrating the skin and enhancing the skin barrier function. This is particularly important in diabetes, as the condition is associated with dry skin and impaired skin barrier function. By using emollients effectively, individuals with diabetes can reduce the risk of skin breakdown and the incidence of skin complications [22-23].

Emollients provide much-needed hydration to the skin, preventing dryness and relieving symptoms such as itching (pruritus). Dry skin is more prone to cracking and infections, leading to serious complications. Regular use of emollients helps maintain the skin's moisture balance and protects it from external irritants. By keeping the skin moisturized, emollients promote healing and reduce the risk of skin-related issues [26,27].

In addition to emollients, topical treatments are valuable in managing diabetic skin complications. Bacterial and fungal infections are common in individuals with diabetes and can exacerbate skin problems. Antibiotics and antifungal medications are often prescribed to treat such infections and prevent their spread. Addressing these infections promptly with topical treatments minimizes the risk of complications and further skin damage [26,27].

Topical steroids are another category of treatment used in diabetic skin care. They are commonly employed to manage itching and inflammation associated with various skin complications. However, it is important to exercise caution when using topical steroids, as prolonged and excessive use can lead to side effects such as skin atrophy. Healthcare professionals should closely monitor the application and dosing of topical steroids to ensure their safe and effective use [26,27].

It is crucial to emphasize that the selection of emollients and topical treatments should be based on the specific type and severity of the skin complication. Different skin conditions may require different approaches and medications. Consulting with a healthcare professional is essential to determine the most appropriate treatment plan and ensure proper application and dosing [25].

The effectiveness of emollients and topical treatments in managing skin complications in diabetes highlights their crucial role in promoting skin health and reducing the morbidity associated with this condition. By incorporating these interventions into a comprehensive skin care regimen, individuals with diabetes can maintain healthy skin, prevent complications, and improve their overall quality of life [26,27].

## Conclusions

Skin complications in diabetes are a significant concern, with diabetic dermopathy, skin infections, and xerosis being some of the most common issues patients face. Several risk factors were identified, including poor glycemic control, obesity, and age. The literature review also highlighted the importance of regular skin care in preventing and managing skin complications and the role of emollients and other topical treatments. The implications for clinical practice are significant, as healthcare providers should be aware of the risk factors and appropriate management strategies for skin complications in diabetes. Regular skin exams and patient education on self-care are also critical components of clinical practice. Future research should focus on developing new and innovative prevention and management strategies for skin complications in diabetes. This may include using new technologies, such as telemedicine and mobile health applications, to enhance patient education and self-care. Additionally, more research is needed to understand the pathophysiology of skin complications in diabetes and identify new treatment therapeutic targets.
